# Historical perspective of aspirin: A journey from discovery to clinical practice Ancient and modern history

**DOI:** 10.34172/jcvtr.2021.28

**Published:** 2021-04-17

**Authors:** Aysa Rezabakhsh, Ata Mahmoodpoor, Hassan Soleimanpour

**Affiliations:** ^1^Cardiovascular Research Center, Tabriz University of Medical Sciences, Tabriz, Iran; ^2^Department of Anesthesiology, Tabriz University of Medical Sciences, Tabriz, Iran; ^3^Emergency Medicine Research Team, Tabriz University of Medical Sciences, Tabriz, Iran

Acetylsalicylic acid (ASA), a synthetic derivative of salicylic acid, is known as a commonly used pharmaceutical agent for over one hundred years. The primary use of salicylic acid refers to the Sumerians, who discovered the analgesic property of the willow plant for the first time. After a long time, around 4000 BC, the analgesic and antipyretic effects of the willow’s leave were also well-known among the Assyrians and the Egyptians.^1^ Moreover, around 1300-1500 BC, some therapeutic effects of the willow and myrtle extractions, were found by Egyptians, to use for colic, gout, and earache treatments. In 1824, the bioactive components of willow bark were completely extracted by two Italian pharmacists, and four years later, the main component of willow, salicin, was isolated. Following the successful extraction of the salicylic acid, in 1853, Frédéric Gerhardt was the first person exposed the acetyl chloride with sodium salicylate to synthesize ASA. ^1^ In 1874, Friedrich von Heyden decided to produce the synthetic form of salicylic acid in his own company, which seemed to be more economical. However, the mass production of salicylates was extended by two founders Friedrich Bayer and William Weskott in dye manufacture, to change its destination from the chemical dye productions to the pharmaceutical targets. A few years later, in 1897, Felix Hoffmann, a German chemist, modified the chemical structure of salicylic acid to synthesize a derivative without any side effect.^2^ Ultimately, by acetylating of the salicylic acid, he developed the conventional used ASA, named “Aspirin”, in which the letter *A* is originated from *acetyl chloride* and the term *spirin* points to the genus name of *Spiraea ulmaria* plant as an enriched source of salicylic acid. Finally, aspirin was patented in the U.S. and then the original powdered form of aspirin was available as a stamped tablet in 1900 and 1904, respectively.^2^

Besides the therapeutic impacts of ASA regarding the coronary thrombosis, angina pectoris, and vascular diseases, the extra-cardiovascular benefits of ASA administration in treatment of various cancers, inflammatory disorders (*e.g.,* Kawasaki diseases, acute rheumatic fever, and pericarditis), preeclampsia, HIV (human immunodeficiency virus), as well as COVID-19 (Coronavirus disease 2019) have been also well-established. Similarly, Craven confirmed the administration of ASA to prevent effectively the recurrent MI and cerebrovascular events, as well.^3^ Despite these advantages, some side effects such as GI bleeding, worsening the asthma, Reye’s syndrome, intracranial hemorrhage, tinnitus, and drug interactions most likely with other NSAIDs have been also reported. In 1975, Samuelsson, a Swedish biochemist, indicated that aspirin could exert an antiplatelet effect by inhibition of thromboxane A2.^4^ In line with this finding, cyclooxygenase isoenzymes were also isolated, which are mainly responsible for prostanoids synthesis from arachidonic acid that can be irreversibly inhibited by aspirin.

## History of clinical practices

**Figure 1:**
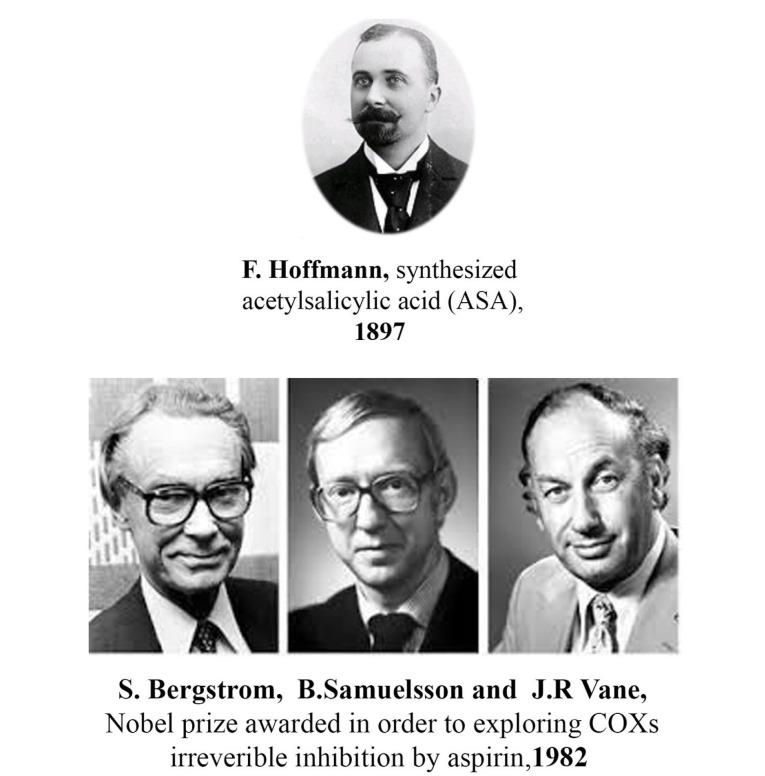
A legendary imagerepresents the scientists who play an important role in aspirin discovery and development.

In 1974, Peter Elwood, an epidemiologist at the UK Medical Research Council, designed the first randomized controlled trial about the potential therapeutic effect of aspirin (300 mg/day) in 1,239 patients with myocardial infarction (MI). the results showed a significant reduction in total mortality, by 12%, and 25% after 6 and 12 months, respectively.^5^ In 1980, a meta-analysis of six randomized clinical trials also revealed that aspirin could decrease the risk of re-infarction by 21% in 10,000 patients with MI. In the second International Study of Infarct Survival (ISIS-2) trial, the impact of aspirin alone (162 mg/day) and in combination with the intravenous streptokinase (1.5 million IU) was evaluated on 17,187 patients for the reduction of mortality and the recurrence events after acute myocardial infarction (AMI). ^6^ The results indicated a relative risk reduction in patients with re-infarction up to 23% in aspirin and 42% in combination group, as well.^6^ The importance of these findings prevailed in 1982 when Vane awarded the Nobel Prize in the field of medicine and shared it with both B. Samuelsson and S. Bergstrom (Figure 1). Together, these benefits were led to the Food and Drug Administration’s approval in 1985, for both treatment and the secondary prevention of AMI. According to a published result of ASPREE (Aspirin in Reducing Events in the Elderly) clinical trial, administration of aspirin in healthy elderly people could not prolong the disability-free survival rate, while led to a higher rate of major hemorrhage than placebo; therefore, in early 2019, the American Heart Association and American College of Cardiology issued a declaration to modify the clinical practice guidelines, regarding the routine administration of aspirin in the elderly subgroup (≥ 70 years), who do not have underlying cardiovascular disease but are at the higher risk for bleeding.

## Competing interest

The authors declared that they have no conflict of interest regarding this study.

## Ethical approval

Not applicable.

## Funding

Not applicable.
